# Novel strategy for food safety risk management and communication: Risk identification for benzoic acid residues in pickled vegetables

**DOI:** 10.1002/fsn3.1839

**Published:** 2020-08-27

**Authors:** Ding‐Yan Lin, Cheng‐Han Tsai, Ying Huang, Siou‐Bang Ye, Che‐Hsuan Lin, Ku‐Yuan Lee, Min‐Hua Wu

**Affiliations:** ^1^ Institute of Food Safety Management National Pingtung University of Science and Technology Pingtung Taiwan; ^2^ Chiayi County Health Bureau Laboratory Section Chiayi Taiwan; ^3^ College of Intelligence National Taichung University of Science and Technology Taichung City Taiwan

**Keywords:** benzoic acid, Raman, risk management, spectral analysis

## Abstract

Benzoic acid (BA) is widely used as an antimicrobial preservative to prolong the shelf‐life of pickled vegetables. A method for rapidly determining the BA content in forty pickled vegetable samples was developed by coupling ultrasonic extraction with surface‐enhanced Raman scattering (SERS) and an adaptive iteratively reweighted penalized least‐squares (AirPLS) algorithm. The results obtained with this method were compared and correlated with those from high‐performance liquid chromatography measurements. Amplification of the Raman scattering via the SERS effect was induced by gold nanoparticles (AuNPs) when BA was irradiated with a 785 nm laser. The AirPLS algorithm was used to reduce the background interference signal, which was also amplified. The amplified Raman scattering effect of BA in the pickled vegetables displayed a positive and significant correlation with the HPLC concentration of BA, with high reproducibility. For HPLC determination of the concentration of BA in the range of 0–820 ppm, the BA monomer's intensity of the 944–1,005 cm^−1^ and 1,366–1,373 cm^−1^ peaks, and BA dimer's intensity of the 1,025 cm^−1^ and 1,465–1,482 cm^−1^ peaks in the SERS spectrum were respectively converted to the *Z*‐ratio BA monomer and *Z*‐ratio BA dimer standard scores by *Z*‐Score conversion. The sum's (*Z*‐ratio BA monomer + *Z*‐ratio BA dimer) sensitivity was 100%, and specificity was 90.9% by receiver operating characteristic curve. This study found that a Raman spectroscopy‐based monitoring method can be one of the fastest screening inspection options that can complete an analysis within a short period of time and produce reliable results. This approach is particularly cost‐effective, which makes it suitable for the initial screening of raw materials and provides an effective management strategy easy to communicate with food safety officials.

## Graphical abstract

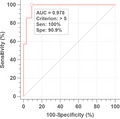

## 1 INTRODUCTION

Internationally, limited funding and manpower have long been the challenge that weaken the ability of food safety officials to deal with issues, especially with frequent occurrences of potentially risk substances. These blind spots in food safety risk management are related to the high costs associated with getting creditable testing results. This high‐cost, long wait‐time practice subsequently hinders many businesses from acquiring adequate certification to verify and validate the safety of their products, let alone the ability to pass highly scrutinized inspection processes. The situation becomes much husher when it comes to small vintage producers, as many are farmers operating on‐farm with limited budget and resources to go through the rigorous food safety inspection process before their products can be allowed to sell on the market.

Therefore, there is a dire need to develop a rapid and cost‐effective technique that could deliver accurate and concise outcomes for the collection and analysis of data for quick detection of potential food safety hazards and risk factors (Chapman & Gunter, 2018). However, existing food testing and certification laboratories have their limitations (Liu, Lu, Huang, Li, & Xu, 2018). For example, it is difficult to verify that the test samples are the foods available on the market. In many cases, the test samples are free of hazardous substances, but the actual food products are noncompliant. In addition, much time is needed to train operators in federal‐level food safety testing laboratories. All of these factors contribute to hindering the development of rapid food safety monitoring systems, especially in remote areas or regions with relatively scarce inspection resources (Wu et al., 2017) pickled vegetables frequently consumed in rural areas, but also served commonly as side dishes in the urban food culture. The presence of benzoic acid (BA) preservatives is often detected in pickled vegetables (Ling et al., 2015). Excessive preservatives can disrupt the normal metabolism of the human body, damage the liver, and even cause cancer. According to the Joint FAO/WHO Expert Committee on Food Additives (JECFA), the acceptable daily intake (ADI) of BA and benzoates is 0–5 mg kg^−1^. However, in highly susceptible populations, even with an ADI of less than 5 mg kg^−1^ (body weight), pseudoallergy or increased hyperactivity in children has been reported (McCann et al., 2007; Piper & Piper, 2017).

To date, techniques such as HPLC, gas chromatography (GC), UV‐visible spectrophotometry, and nuclear magnetic resonance have been used to detect BA (Asensio‐Ramos, Hernández‐Borges, Rocco, & Fanali, 2009; Cordella, Moussa, Martel, Sbirrazzuoli, & Lizzani‐Cuvelier, 2002; Schieberle & Molyneux, 2012). Nonetheless, these methods are time consuming and require expensive equipment when performing pretreatments and are not effective for rapid food safety monitoring tasks (Dong, Mei, & Chen, 2006; Pylypiw & Grether, 2000; Techakriengkrai & Surakarnkul, 2007). What the market needs are a method with rapid pass‐through time to serve as a prescreening step determining whether the samples need to undergo more scrutiny by technologies such as HPLC. This is particularly crucial when it comes to risk management and food safety monitoring practices.

From prior investigations, we have identified the prospective utility of surface‐enhanced Raman scattering (SERS) for the rapid monitoring of BA in pickled foods (Tsen et al., 2019; Xue et al., 2020). The purpose of this study is to develop a SERS‐based method for the rapid detection of BA in pickled vegetables that is inexpensive, convenient, effective, and only uses small pretreatment equipment to enable field monitoring. It was expected that this type of rapid screening will become an integral part of any food safety management and communication scheme. By leveraging a quick and economical “screen first, test later” strategy, it becomes feasible for the regulatory authorities to incorporate a more public‐oriented approach for managing and disseminating information on food safety risks.

## 2 MATERIALS AND METHODS

### 2.1 Samples

A cross‐sectional study was conducted from May 2019 to December 2019. Forty pickled vegetable samples were obtained from the retail market in Chiayi County, Taiwan, including 15 pickled radishes, 19 bamboo shoots, 5 pickled cabbages, and 1 tender ginger. The 40 pickled vegetable samples were collected, placed in sealed bags, and refrigerated in a refrigerator at 4°C to prevent sample contamination. All of the pickled vegetable samples were processed and analyzed by using the same pretreatment equipment and SERS and HPLC instruments. The analysis conditions are described below.

### 2.2 Pretreatment

The pickled vegetables were stirred and thoroughly mixed, after which 5 g of each sample was accurately weighed. Thereafter, 30 ml of a 50% methanol solution was added to the samples. After 10 min of sonication, the samples were centrifuged at 1,076 *g* to retrieve the extract. Subsequently, 30 ml of a 50% methanol solution was added, and the samples were sonicated for 10 min and centrifuged at 1,076 *g* to retrieve the extract. Methanol (10 ml) was then added to the above solution (30 + 30 + 30 = 90 ml) to a specified volume of 100 ml. An appropriate amount of extract was filtered through a No. 1 filter paper to produce 50 ml of filtrate. Finally, the supernatant from filtration was passed through a PVDF syringe filter with a pore size of 0.22 μm and a 13 mm PVDF membrane filter and collected as the test solution.

### 2.3 Surface‐enhanced raman spectroscopy (SERS) detection

The aforementioned test solution (4 µl) was dispensed onto a SERS substrate (Phan2 SERS) produced by Phansco Co., Ltd., Taiwan. The substrate contained AuNPs (average diameter = 55 nm) for Raman enhancement. The SERS substrate was placed in a commercial handheld SERS spectrometer (DeltaNu Inspector Raman, USA) for evaluation of the BA spectrum. The laser emission wavelength was 785 nm, the laser power was 120 mW, and the system resolution was 8 cm^−1^. The acquisition time for the SERS data was set to 1 s, and all measurements were performed at room temperature. The measurement wavelength range was 204–1,986 cm^−1^, and a total of 1,024 peaks were recorded.

### 2.4 Formulation of standard BA solution

Analytical grade BA (C_6_H_5_COOH) was purchased from Sinopharm Chemical Reagent Co., Ltd. BA was dissolved in 0.1 N sodium hydroxide solution by ultrasoniction, and deionized water was added to a specified volume of 100 ml to prepare the standard BA solution with a concentration of 500 ppm. The ultrapure water was acquired from Simplicity Water Purification Systems (Millipore, Molsheim, France).

### 2.5 AirPLS algorithm

The AirPLS algorithm can be applied to correct the baseline drift phenomenon in SERS in order to improve the smoothness of the baseline signal (Zhang, Chen, & Liang, 2010). The AirPLS algorithm iteratively changes the weights of the sum of squares error (*SSE*) between the fitted baseline and the BA signals. The weights of the *SSE* were obtained from the difference between the preadaptive fitted baseline and the BA signals. The algorithm is composed of two parts: smoothing of the signals by the least‐squares algorithm and adaptive iteration, which transforms the process of the least‐squares algorithm for baseline estimation. The AirPLS algorithm was executed with SpectraView® software.

### 2.6 High‐performance liquid chromatography (HPLC) detection

The pretreatment test solutions of the 40 samples were commissioned to Chiayi County Health Bureau for testing in accordance with the HPLC method for BA testing published by Kishi and coworkers (Kishi & Yamada, 2007). The 40 samples preparation procedure was as simple as possible. Analytical separation was carried out using L‐2000 machine HPLC system (Hitachi, Japan) equipped with a L‐2130 pump and L‐2455 diode array detector. An HPLC Inertsil ODS‐2 column (150 mm × 6.0 mm, 5 μm) from GL Sciences was selected for the separation. The injection volume was 10 µl and the column oven temperature was set at 35 degrees Celsius. The mobile phase was methanol/acetonitrile/5 mM citric acid buffer (pH = 4.3) (1:2:7, v/v/v); the flow rate was 1.0 ml/min. A photo diode array detector was used and the detection wavelength was set at 230 nm for benzoic acid.

### 2.7 Definition and specification of results

**FIGURE 1:**
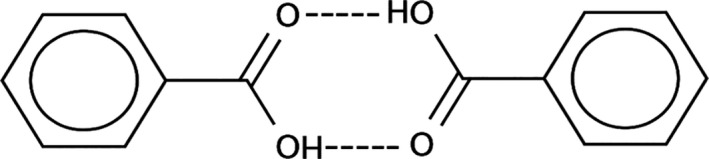
Dimer Effect of Benzoic Acid in Liquids

**FIGURE 2:**
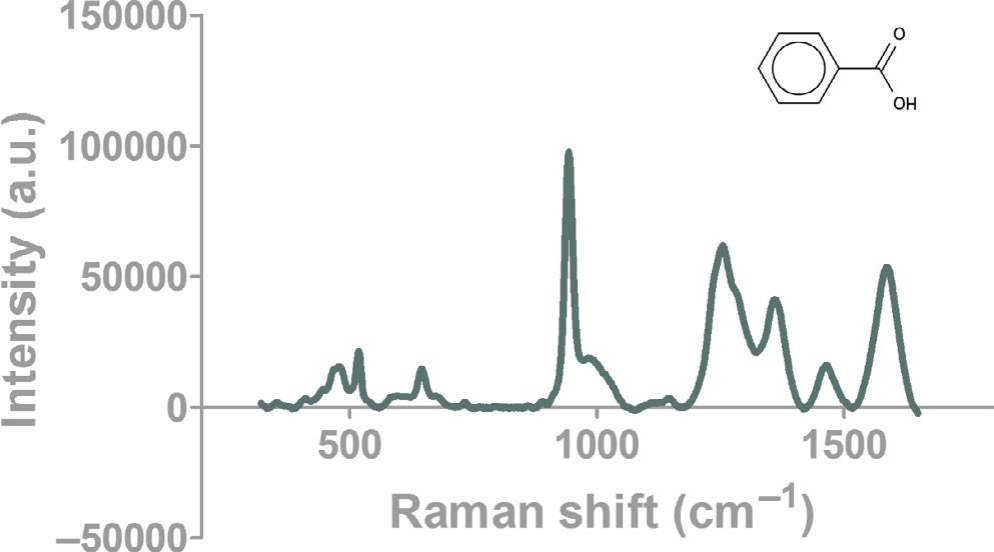
Raman spectroscopy of BA monomer bound to SERS nanogold

**FIGURE 3:**
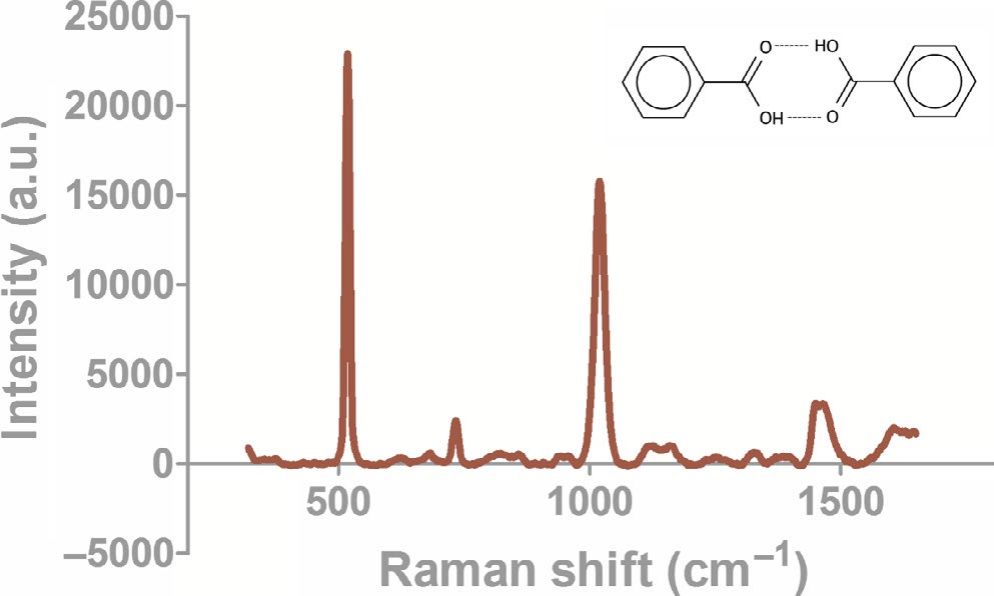
Raman spectrograms of BA dimer and SERS nanogold combination

The SERS‐AirPLS spectrum of BA obtained in this study is shown in Figures 1, 2 and 3. In addition to being present as a BA single molecule, benzoic acid also appears as a dimer in the extraction solution. Besides, a single benzoic acid molecule (BA monomer) and a benzoic acid dimer molecule (BA dimer) are also present in the extract. Binding to nanogold produces peaks at different Raman wavelengths. When the BA monomer and the BA dimer combine at different angles to the nanogold, the emergence point of the Raman peak is drifted (Shift) effect, so SERS‐AirPLS needs to capture the benzoic acid signal with a long Raman interval. As shown in Table 1, the Raman peaks of the BA monomer were located at 944–1,005 cm^−1^ and 1,366–1,373 cm^−1^. The Raman peaks of the BA dimers were located at 1,025 cm^−1^ and 1,465–1,482 cm^−1^. In this study, two intervals at the BA’s monomer and dimer in the SERS‐AirPLS spectrum were selected as the peaks for BA identification. The SERS‐AirPLS peak at 944–1,005 and 1,025 cm^−1^ represents the naphthenic structure of BA’s monomer and dimers, and the SERS‐AirPLS peak at 1,366–1,373 and 1,465–1,482 cm^−1^ represents the structure of the carboxylic acid functional group of BA. When significant fluctuations were observed at both intervals in the SERS‐AirPLS spectrum, the pickled vegetable samples were determined to contain BA.

**TABLE 1:** Reading Interval of Benzoic Acid in SERS Signal

BA	naphthenic structure	carboxylic acid functional group
Monomer	944–1,005	1,366–1,373
Dimer	1,025	1,465–1,482

### 2.8 Data collection and verification

The definition of a significant fluctuation is as follows: after applying the AirPLS algorithm to smooth the baseline signal of the SERS spectra of the 40 pickled vegetables, the peak intensity values at 944–1,005 cm^−1^, 1,366–1,373 cm^−1^, 1,025 cm^−1^, and 1,465–1,482 cm^−1^ for each of the 40 pickled vegetable samples were converted to the statistical *Z*‐score based on the intensity distribution of their individual 1,024 SERS‐AirPLS peaks. Thereafter, *Z*‐Score_naphthenic structure_ was divided by *Z*‐Score_carboxylic acid functional group_ to obtain the *Z*‐Ratio. If the sum of *Z*‐ratio of the peak intensity at 944–1,005 cm^−1^, 1,366–1,373 cm^−1^, 1,025 cm^−1^, and 1,465–1,482 cm^−1^ was greater than or equal to 1.5, this is defined as a significant fluctuation.

### 2.9 Statistical method and software

Statistical analysis and the aforementioned *Z*‐Score calculations were performed using the Medcalc statistical software (Version 19.2; MedCalc, Ostend, Belgium). The receiver operating characteristics (ROC) curve was used to determine the optimal *Z*‐Ratio for identifying concentrations of BA that exceeded the regulatory standard of 600 ppm. The optimal value was defined at the tangent point of the ROC curve, where the sensitivity was 100%, and the specificity was at the maximum. This study employed Monte Carlo simulations to analyze the risk of pseudo‐positive and pseudo‐negative of ROC’s results between the concentration of BA in the 40 pickled vegetables.

## 3 RESULTS

From previous literature, different Raman peaks were selected to confirm the presence of BA. For example, the peak of the naphthenic structure appeared at 944–1,005 cm^−1^ and 1,025 cm^−1^ in the present study, whereas this peak appeared at 994 cm^−1^ in a prior study (Cai, Dong, Wang, & Chen, 2018). The difference in the peak frequency originates from the different SERS materials. In the 2018 study, pure gold was used as the metal medium for enhancement; in this study, silver–gold core–shell nanoparticles were used in the preparation process.

In the present study, the BA content of the 40 pickled vegetables was also assayed by HPLC, as listed in Table 2. The pickled vegetables included 15 pickled radishes, 19 bamboo shoots, 5 pickled cabbages, and 1 tender ginger, and their average BA concentration ± standard deviations were 409 ± 277 ppm, 50.0 ± 150 ppm, 62.0 ± 58.9 ppm, and 0.00 ± 0.00 ppm, respectively. The minimum and maximum BA concentration in the 40 pickled vegetables were 0 ppm and 820 ppm, respectively.

**TABLE 2:** *Z*‐score at Raman peak of BA monomer and dimer compared with HPLC

No.	A (*Z*‐ratio of BA monomer)	B (*Z*‐ratio of BA dimer)	A + B (Sum of *Z*‐ratio)	HPLC
1	1.58	6.27	7.85	820
2	0.00	7.81	7.81	690
3	4.88	3.58	8.46	650
4	3.33	4.88	8.21	590
5	5.02	5.30	10.3	570
6	5.12	0.00	5.12	560
7	6.50	0.00	6.50	510
8	5.00	0.00	5.00	490
9	0.00	5.32	5.32	480
10	0.00	7.82	7.82	470
11	4.62	0.00	4.62	440
12	4.46	0.00	4.46	440
13	0.00	5.56	5.56	380
14	−0.8	0.00	−0.8	130
15	−0.32	0.00	−0.32	120
16	0.65	0.00	0.65	30
17	−15.7	0.00	−15.7	30
18	0.67	0.00	0.67	0
19	0.69	0.00	0.69	0
20	0.64	0.00	0.64	0
21	0.67	0.00	0.67	0
22	0.91	0.00	0.91	0
23	1.20	0.00	1.20	0
24	0.86	0.00	0.86	0
25	1.21	0.00	1.21	0
26	0.87	0.00	0.87	0
27	0.91	0.00	0.91	0
28	1.14	0.00	1.14	0
29	0.65	0.00	0.65	0
30	0.42	0.00	0.42	0
31	0.96	0.00	0.96	0
32	0.81	0.00	0.81	0
33	0.81	0.00	0.81	0
34	0.59	0.00	0.59	0
35	1.38	0.00	1.38	0
36	1.07	0.00	1.07	0
37	−0.25	0.00	−0.25	0
38	1.04	0.00	1.04	0
39	0.65	0.00	0.65	0
40	1.19	0.00	1.19	0

**FIGURE 4:**
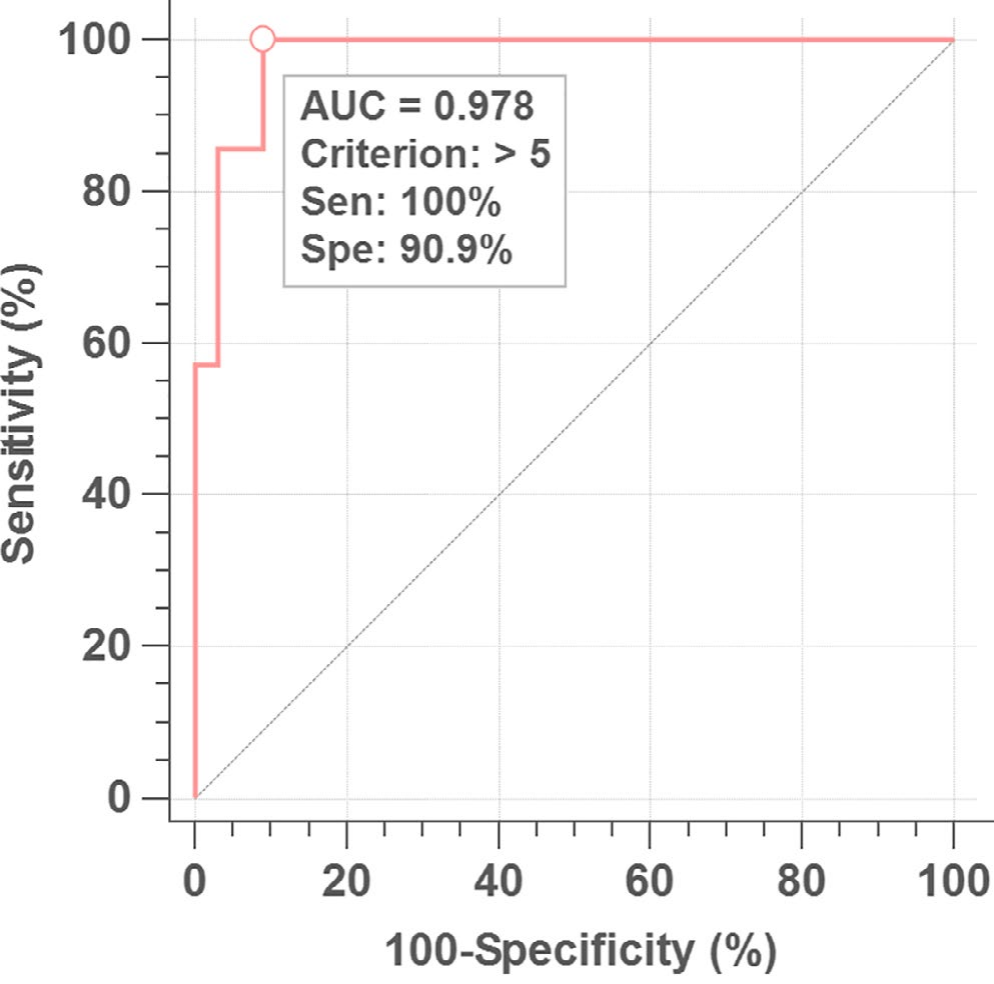
When the Raman Sum of *Z*‐ratio is >5, it can be judged as a high‐risk sample under Raman spectroscopy, and when the Raman Sum of *Z*‐ratio is <5 (ROC), it can be judged as a low‐risk sample

The purpose of this study is to determine whether the SERS‐AirPLS spectrum is adequate as a rapid‐screening instrument for pickled vegetables. Table 2 shows the descriptive statistical information of the *Z*‐ratio and summation and HPLC collected for 40 samples under the rule of Table 1. There are two types of benzoic acid in pickled vegetables. One is monomolecular, and the other is dimeric, and these two types can be different angles from each other to combine gold nanoparticles (AuNPs) (Gao, Hu, Li, Zhang, & Chen, 2013). Neither monomers nor dimers of benzoic acid were observed to bind more readily to the gold nanoparticles. However, as the concentration of benzoic acid gradually increased, it was easier to observe the appearance of BA dimers in this study. When the concentration of benzoic acid in pickled vegetables has changed, the corresponding Raman Sum *Z*‐ratio also has variation, and Pearson's correlation coefficients are 0.737. The ROC curve analysis in Figure 4 shows that when the sum of *Z*‐ratio is more than 5, the risk of exceeding 600 ppm BA concentration in pickled vegetables will be rising. This criterion which the sum of *Z*‐ratio is more than 5 was named “Rule 5”.

**FIGURE 5:**
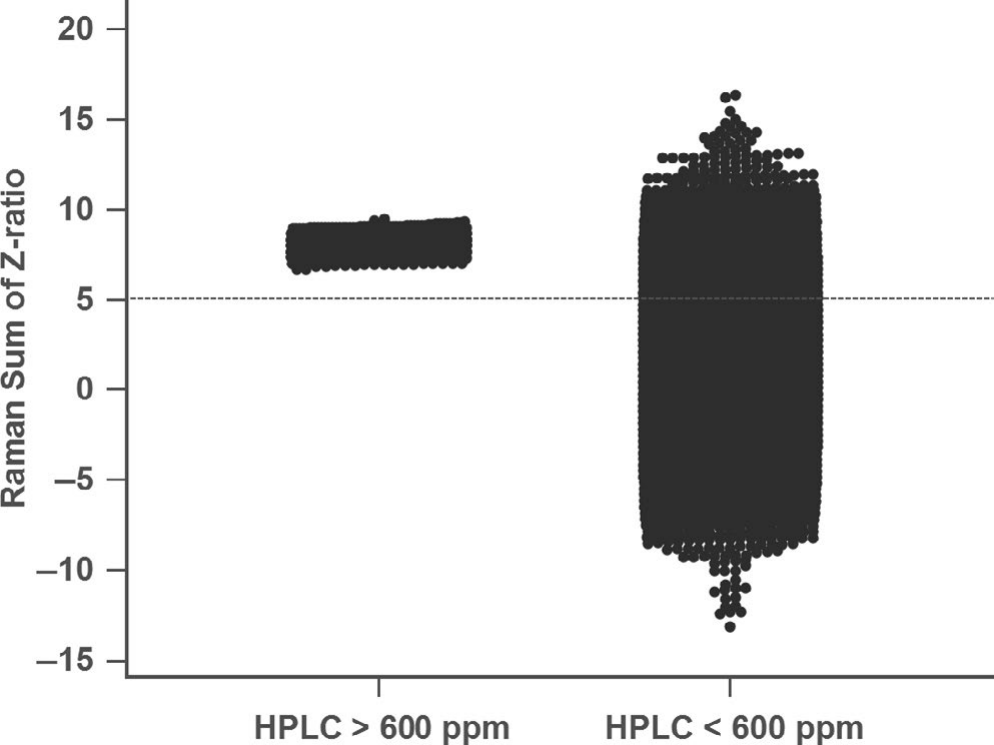
Results from 10,000 Monte Carlo simulations showed that when the HPLC concentration of benzoic acid in the pickled vegetable samples was above 600 ppm, the HPLC concentration of benzoic acid in the pickled vegetable samples was higher than 600 ppm. When using the results in Figure 5, the Raman Sum of *Z*‐ratio was to obtain the large Raman spectral fraction. If the HPLC concentration is less than 5 points, the sample will be detected as exceeding the standard. However, when the HPLC concentration of benzoic acid in the pickled vegetable samples was below 600 ppm, about 20% of the samples needed to be re‐detected. This method has significantly reduced the waste of sending 80% of low‐risk samples to HPLC for testing

In this study, 10,000 Monte Carlo simulations were used to analyze whether Rule 5 had enough power to select pickled vegetable samples with benzoic acid concentrations exceeding 600 ppm. The results showed that by applying Rule 5, it was possible to detect pickled vegetables with benzoic acid levels exceeding the ban (Figure 5). However, when the HPLC concentration of benzoic acid in pickled vegetables is below 600 ppm, about 20% of the samples monitored by "Rule 5" need to be sent to HPLC again. Still, this method has significantly reduced the waste of sending 80% of the low‐risk samples to HPLC for testing.

This study only utilized simple ultrasonic extraction and filter paper filtration with no complicated pretreatment procedures, such as dispersive liquid–liquid microextraction (Xue et al., 2020). The results demonstrate the feasibility of using the SERS‐AirPLS spectrum to identify whether pickled vegetables exceed the regulatory standard of 600 ppm for the task of monitoring and rapid screening. Samples that do not exceed the BA standard can be screened out, which reduces the extra testing costs and time required for HPLC analysis.

The *Z*‐Ratio threshold was then examined to determine which pickled vegetables samples should be subjected to HPLC for accurate analysis by analyzing the ROC. As shown in Figure 4, for the pickled vegetable samples with a *Z*‐Ratio greater than 5 that were sent for HPLC analysis, the area under the curve (AUC) was 0.978, which indicated outstanding discrimination. Moreover, the sensitivity was 100%, and the specificity was 90.9%. Under the conditions mentioned above, the SERS‐AirPLS spectrum could be used to identify all samples that exceeded the regulatory standard of 600 ppm, but at the same time, there were nearly 9.1% false positives. False positives are acceptable for monitoring and rapid‐screening techniques, whereas false negatives are unacceptable—no false negatives were present in this study.

## 4 DISCUSSION

Using HPLC analysis of the BA residues in pickled vegetables as the benchmark, it was concluded that the developed SERS‐AirPLS technique had a discrimination of 0.978, a sensitivity of 100%, a specificity of 90.9%, and no false negatives results. The results demonstrate the screening practicality of the SERS‐AirPLS technique for monitoring the BA concentration in pickled vegetables.

Previous literature has stated that although Raman spectroscopy is effective for qualitative analysis, quantitation using this method is not fully understood (Porter, Lipert, Siperko, Wang, & Narayanan, 2008). A possible reason for the quantification uncertainty is that in the past, the Raman signals were too weak, and thus the performance at low concentrations was unreliable (Porter et al., 2008). On the other hand, amplification of the Raman signal by SERS, as applied to BA screening herein, produced a signal that was more than 10^14^ times higher than that of traditional Raman spectroscopy (Kneipp, Kneipp, Itzkan, Dasari, & Feld, 1999). For this reason, we hypothesized that SERS could allow semi‐quantitative analysis of BA.

In the past, few studies have investigated the use of Raman spectroscopy for the detection of preservatives in foods. Among the existing studies, the main application was for the detection of BA in carbonated beverages and medicines (Cai et al., 2018; Xue et al., 2020). In these research areas, novel pretreatment methods such as silicone thin film microextraction or dispersive liquid–liquid microextraction were coupled with SERS to determine the concentration of BA. Metal nanoparticles were evenly distributed on the substrate, and the acquired SERS signal of BA was strong with high reproducibility. In comparison, this study attempted to determine whether the concentration of BA residues in pickled vegetables exceeded the 600 ppm threshold by applying SERS‐AirPLS after using only simple ultrasonic extraction and filtration through a No.1 filter paper. This study showed good linearity between the SERS signal intensity at “Sum of *Z*‐ratio” and the BA concentration in the range of 380–820 ppm. The detection limit was in the range of 80 ppm, and limit of quantification was 265 ppm, respectively.

In the past 110 years, food inspection has developed from classical analysis to modern automated analysis, in particular, the application of chromatography and spectroscopy in food analysis (Mejac, Bryan, Lee, & Tran, 2009). However, these methods require longer training time and involve relatively complicated pretreatments. In addition, because the pretreatments are more complicated, these techniques are relatively time consuming and are relegated to analysis in government‐certified laboratories. The biggest challenge is the lack of high‐throughput sample screening capability without an adequate budget.

This study demonstrates that Raman spectroscopy and big data algorithms can be used to detect BA residues in vegetables, and only 11 samples out of the 40 pickled vegetables required HPLC analysis, which significantly reduced the inspection cost and time. Compared with HPLC, SERS has many advantages: first, the signals in the Raman spectra correspond to the molecular structure. Thus, the content of preservatives in pickled vegetables can be identified without extensive extraction or ionization of the sample and special labeling of the test compound. Second, although pickled vegetables still contain 20%–40% water, the Raman signal of water is very weak (water is a polar substance), and pickled vegetables can be tested directly without special dehydration treatment. Compared with the infrared spectral analysis, which has been widely used in the past, SERS is more suitable for real‐time field detection of BA. Because SERS requires only a small amount of sample (microliters), it can provide cheap, simple, portable, fast, and sensitive analysis of BA residues in pickled vegetables. This detection method is the first application of its kind for the detection of BA residues in pickled vegetables with 20%–40% water activity.

## 5 CONCLUSIONS

In the last 20 years, while screening benzoic acid in complex true food samples, many scientists have not only made every effort to follow the protocol established HPLC as practiced but also has developed the use of FTIR as alternative problem‐solving techniques. However, food is a kind of full water material. In the goal of enabling field monitoring for food, HPLC and FTIR are not good choices, because HPLC is inconvenient and FTIR needs a dehumidifier. SERS‐based methods show that we have adapted well to the environment we work and have developed our unique strategies and solutions with a high level of excellence. In this regard, we believe that the SERS‐based methods will play an essential role in improving our standards of practice in screening BA. Moreover, this approach is particularly cost‐effective, which makes it suitable for the initial testing of raw material and provides an alternative management and communication strategy for food safety risks.

## CONFLICTS OF INTEREST

The authors have declared no conflict of interest.

## ETHICAL STATEMENT

The authors declare that this study did not involve human or animal subjects and human and animal testing are unnecessary in our study.

## INFORMED CONSENT

Written informed consent was obtained from all participants.
